# Supplementary material for: Analysis of PPARγ Signaling Activity in Psoriasis
Source: Int J Mol Sci. 2021 Aug 10;22(16):8603. doi: 10.3390/ijms22168603 (PMC8395241; doi:10.3390/ijms22168603)
Supplement: Supplementary file 1 [file ijms-22-08603-s001.zip › Supplemental materials_Analysis of PPARg signaling activity in psoriasis/Pathway models/Models images and html files/Model of PPARG signaling in psoriasis/13434.html]

IL1RL2


# Protein IL1RL2

|  |  |
| --- | --- |
| URN | urn:agi-llid:8808 |
| Total Entities | 0 |
| Connectivity | 275 |
| Name | IL1RL2 |
| Description | interleukin 1 receptor like 2 |
| Notes | The protein encoded by this gene is a member of the interleukin 1 receptor family. An experiment with transient gene expression demonstrated that this receptor was incapable of binding to interleukin 1 alpha and interleukin 1 beta with high affinity. This gene and four other interleukin 1 receptor family genes, including interleukin 1 receptor, type I (IL1R1), interleukin 1 receptor, type II (IL1R2), interleukin 1 receptor-like 1 (IL1RL1), and interleukin 18 receptor 1 (IL18R1), form a cytokine receptor gene cluster in a region mapped to chromosome 2q12. [provided by RefSeq, Jul 2008] |
| Primary Cell Localization | Plasma membrane |
| Class | Receptor |

---

|  |  |
| --- | --- |
| Pathway | IL36 Receptor Antagonist (IL1F5) Deficiency (DITRA) |
|  | Model of PPARG signaling in psoriasis |

---

|  |  |
| --- | --- |
| MedScan ID | 8808 |

---

|  |  |
| --- | --- |
| LocusLink ID | 8808 |
|  | 107527 |
|  | 171106 |

---

|  |  |
| --- | --- |
| Alias | IL1RL2 |
|  | interleukin IL1 receptor-like II |
|  | IL-36 receptor |
|  | LOC113807 |
|  | interleukin-1 receptor-related protein 2 |
|  | interleukin IL1RRP2 |
|  | interleukin IL1 receptor-related protein 2 |
|  | IL1R-RP2 |
|  | interleukin IL1RL2 |
|  | interleukin IL1R-RP2 |
|  | interleukin IL1 receptor-like 2 |
|  | AI481289 |
|  | interleukin 1 receptor-like 2 |
|  | interleukin 1 receptor-like II |
|  | interleukin IL1 receptor-related protein II |
|  | AW551444 |
|  | IL1F8 receptor |
|  | IL-36R |
|  | IL1RRP2 |
|  | LOC115605 |
|  | interleukin 1 receptor related protein two |
|  | interleukin-1 receptor-related protein II |
|  | IL-1 receptor-related protein 2 |
|  | IL-1Rrp2 |
|  | interleukin-1 receptor-like 2 |
|  | interleukin-1 receptor related protein 2 |

---

|  |  |
| --- | --- |
| GO ID | 0004908 |
|  | 0004909 |
|  | 0006968 |
|  | 0071345 |
|  | 0019221 |
|  | 0006954 |
|  | 0045087 |
|  | 0070498 |
|  | 0045582 |
|  | 0032755 |
|  | 0050727 |
|  | 0007165 |
|  | 0005887 |
|  | 0005886 |
|  | 0004896 |
|  | 0002376 |
|  | 0016021 |
|  | 0016020 |

---

|  |  |
| --- | --- |
| KEGG ID | hsa:8808 |
|  | mmu:107527 |
|  | rno:171106 |

---

|  |  |
| --- | --- |
| Organism | Homo sapiens {Organism urn:agi-taxid:9606} |
|  | Mus musculus {Organism urn:agi-taxid:10090} |
|  | Rattus norvegicus {Organism urn:agi-taxid:10116} |

---

|  |  |
| --- | --- |
| Mouse chromosome position | 1 |

---

|  |  |
| --- | --- |
| OMIM ID | 604512 |

---

|  |  |
| --- | --- |
| Rat chromosome position | 9q22 |

---

|  |  |
| --- | --- |
| Hugo ID | 5999 |

---

|  |  |
| --- | --- |
| Human chromosome position | 2q12.1 |

---

|  |  |
| --- | --- |
| Swiss-Prot Accession | Q9HB29 |
|  | Q9HB29.2 |
|  | Q149G7 |
|  | Q9ERS7 |
|  | Q9ERS7.1 |
|  | Q62929 |
|  | G3V7W4 |
|  | Q62929.1 |
|  | A4FU63 |
|  | Q13525 |
|  | Q45H74 |
|  | Q53TU8 |
|  | Q587I8 |

---

|  |  |
| --- | --- |
| PIR ID | G02426 |

---

|  |  |
| --- | --- |
| GenBank ID | NC\_000002 |
|  | XR\_923053 |
|  | NM\_001351447 |
|  | NP\_001338376 |
|  | NM\_001351448 |
|  | NP\_001338377 |
|  | XM\_017005174 |
|  | XP\_016860663 |
|  | XM\_011512092 |
|  | XP\_011510394 |
|  | XM\_011512096 |
|  | XP\_011510398 |
|  | XM\_011512091 |
|  | XP\_011510393 |
|  | XM\_011512093 |
|  | XP\_011510395 |
|  | NM\_001351449 |
|  | NP\_001338378 |
|  | NM\_003854 |
|  | NP\_003845 |
|  | NM\_001351450 |
|  | NP\_001338379 |
|  | XM\_017005175 |
|  | XP\_016860664 |
|  | XM\_017005176 |
|  | XP\_016860665 |
|  | XM\_006712820 |
|  | XP\_006712883 |
|  | XM\_017005177 |
|  | XP\_016860666 |
|  | NM\_001351446 |
|  | NP\_001338375 |
|  | XM\_011512094 |
|  | XP\_011510396 |
|  | XM\_017005180 |
|  | XP\_016860669 |
|  | XM\_006712822 |
|  | XP\_006712885 |
|  | AC007248 |
|  | AAY15046 |
|  | AC007271 |
|  | AAX81989 |
|  | CH471127 |
|  | EAX01798 |
|  | DQ131903 |
|  | AAZ38712 |
|  | AF284434 |
|  | AAG21368 |
|  | AK315334 |
|  | BAG37734 |
|  | BC107064 |
|  | AAI07065 |
|  | CA448166 |
|  | U49065 |
|  | AAB53237 |
|  | Q9HB29 |
|  | NC\_000067 |
|  | XM\_006495608 |
|  | XP\_006495671 |
|  | NM\_133193 |
|  | NP\_573456 |
|  | XM\_006495611 |
|  | XP\_006495674 |
|  | XM\_006495609 |
|  | XP\_006495672 |
|  | XM\_006495610 |
|  | XP\_006495673 |
|  | NM\_001356478 |
|  | AC167168 |
|  | CH466536 |
|  | EDL14573 |
|  | EDL14574 |
|  | AF284433 |
|  | AAG21367 |
|  | AK054530 |
|  | AK085780 |
|  | BC004628 |
|  | AAH04628 |
|  | BC117797 |
|  | AAI17798 |
|  | Q9ERS7 |
|  | NC\_005108 |
|  | XM\_017596258 |
|  | XP\_017451747 |
|  | XM\_017596260 |
|  | XP\_017451749 |
|  | XM\_017596259 |
|  | XP\_017451748 |
|  | NM\_133575 |
|  | NP\_598259 |
|  | AC\_000077 |
|  | AABR07067459 |
|  | AABR07067460 |
|  | AAHX01059549 |
|  | AAHX01059550 |
|  | AAHX01059551 |
|  | AAHX01059552 |
|  | AAHX01059553 |
|  | CH473965 |
|  | EDL99176 |
|  | EDL99177 |
|  | U49066 |
|  | AAB53238 |
|  | Q62929 |

---

|  |  |
| --- | --- |
| Swiss-Prot ID | ILRL2\_HUMAN |
|  | ILRL2\_MOUSE |
|  | ILRL2\_RAT |

---

|  |  |
| --- | --- |
| Cell Localization | Membrane |

---

|  |  |
| --- | --- |
| Ensembl ID | ENSG00000115598 |
|  | ENSP00000413348.2 |
|  | ENST00000441515.3 |
|  | ENSP00000264257.2 |
|  | ENST00000264257.7 |
|  | ENSMUSG00000070942 |
|  | ENSMUSP00000092630.3 |
|  | ENSMUST00000095020.8 |
|  | ENSMUSP00000142248.1 |
|  | ENSMUST00000194296.5 |
|  | ENSRNOG00000014683 |
|  | ENSRNOP00000019880.1 |
|  | ENSRNOT00000019880.3 |

---

|  |  |
| --- | --- |
| MGI ID | MGI:1913107 |

---

|  |  |
| --- | --- |
| RGD ID | 621782 |

---
